# The London Government Bill

**Published:** 1899-03-11

**Authors:** 


### The London Government Bill.

So far as the creation of distinct municipalities in London will tend to give dignity to the machinery of local government in the metropolis, and will draw a better class of residents in the various parishes to take an interest in the administration of their own districts, Mr. Balfour's London Government Bill deserves to receive the most sympathetic consideration. At the same time we would point out that, admirable as are the examples of municipal government offered for our study and our imitation by some of the great provincial towns, there are certain points in regard to which the position of London is so peculiar that lessons derived from even the largest towns can have but little bearing upon the conditions existing here. In every other municipality, however great the number of the inhabitants, the town is cut off from all other towns by a ring of more or less open country, and, so far as its sanitary administration is concerned, can regard itself as an isolated unit. The new metropolitan boroughs, however, will find themselves in no such enviable position. It will, no doubt, often be the case that one side of a street will be in one town and the opposite side in another, that the lamp-posts may be of different patterns, and even that the two sides of the street may be swept by different men and at different times. Such things do not greatly matter; they furnish food for laughter, and that is all. But in regard to many sanitary affairs, and especially in reference to measures which may be necessary for the prevention of the spread of disease, this interlocking of the different districts is a much more important matter, and it would be well that in the further consideration of the Bill every care should be taken to ensure that harmony shall reign, and that uniformity of practice in sanitary administration shall be maintained among the various districts which will so closely abut upon one another. It is indeed a matter of no small interest to observe that, at the very moment when it is proposed to give a large degree of municipal independence to districts more or less arbitrarily carved out of a thickly inhabited area like London, districts which must of necessity be sharp-edged and strictly circumscribed, many provincial boroughs are becoming impatient at the restrictions imposed upon them, so far as health matters are concerned, by the geographical limits of their sanitary areas, and are asking for their powers to be extended over the neighbouring country. If urban authorities in the provinces find themselves hampered in their endeavours to control infection by the fact that it often comes from the outer zone, over which they can exercise no supervision—find, for example, that the means at their disposal for controlling a suspected source of milk supply are complicated and unsatisfactory, and that they often have no power to protect their own water supplies from pollution—how much more will not the new metropolitan boroughs find themselves hampered if they cannot follow a case of scarlet fever across the street into another parish, and cannot stop the sale of milk from a shop over the way from which infection is being spread throughout their own district? Of course we know that this is no new difficulty, and indeed that at the present time it is in full operation, but that is all the more reason why the matter should receive the careful attention of those who are responsible for the new Bill, and that the opportunity should be taken, when the duties of the new sanitary authorities are being defined, to arrange for the creation of some much more intimate link between their medical officers than has hitherto existed. That each authority should be responsible for the sanitary administration of its own district is only in accordance with the general principle which is at the bottom of the Bill. But what is abundantly clear is that none of these new boroughs can afford to remain indifferent to the sanitary affairs of their neighbours, or, in laying down a sanitary policy, to disregard what is going on around them; and the question arises whether the time has not come when the thirty or more medical officers of health within the metropolitan boundaries might not well be united in one metropolitan medical service. Each sanitary authority would then have its own complete administrative sanitary staff, but its medical adviser would be an officer of the County Council, a member of the general medical board whose head would be the medical officer to the County of London. This would afford the link that is required, would ensure the mutual transference of the information useful to all, and would enable health matters to be dealt with in a broader spirit and in a more effectual manner than is possible so long as the connection of the central office with the district authorities is mainly correctional and disciplinary.

